# Multiparametric robust sensing via readout of characteristic magnetization loops

**DOI:** 10.1038/s41598-026-42763-x

**Published:** 2026-03-03

**Authors:** Michael P. Path, Michael Vogel, Jeffrey McCord

**Affiliations:** 1 Nanoscale Magnetic Materials - Magnetic Domains, Department of Materials Science, Faculty of Engineering, Kiel University, 24143 Kiel, Germany; 2 Kiel Nano, Surface and Interface Science (KiNSIS), Kiel University, 24118 Kiel, Germany

**Keywords:** multiparameter sensing, magnetic hysteresis, magneto-optics, thermal sensing, magnetic sensing, machine learning, Materials science, Optics and photonics, Physics

## Abstract

Simultaneous measurements of multiple physical quantities under variable conditions are essential from fundamental research to application. However, multiparametric sensors often need intricate calibration procedures which can be compromised by changing environmental conditions. Here, we present a robust sensing concept based on the Fourier components of a nonlinear response of an excitation. By extracting amplitude ratios and phase differences between different harmonics, a set of characteristic shape parameters are extracted ensuring independence from external factors such as amplifier gain or instrumental delays. This principle is demonstrated using magneto-optical readout of magnetization loops in a perpendicular bismuth-substituted yttrium iron garnet indicator film. Two-dimensional parameter maps spanning both temperature and magnetic field are measured, providing a sensor-specific fingerprint for parallel detection. Using lookup-table interpolation and a random forest regressor, robust and accurate simultaneous extraction of temperature and magnetic field is demonstrated. The presented magneto-optical method offers a universal, gain and delay invariant framework for multiparametric sensing in nonlinear materials.

**Supplementary Information:**

The online version contains supplementary material available at 10.1038/s41598-026-42763-x.

## Introduction

Simultaneous extraction of multiple physical quantities from a single sensor element, or multifunctional/multiparametric sensing, has gained increasing attention due to its potential in diverse scientific and industrial applications^1^. Such approaches can improve the overall performance^2^, avoid spatial mismatches^3^, and can coordinate between multiple individual sensor systems^1^, especially under varying environmental conditions. A particularly relevant parameter combination is magnetic field and temperature, for example in power electronics^4^. However, calibration is often complex^2^ and has recently also been realized with machine learning^5^. We propose a robust method that exploits the characteristic shape of magneto-optically read out hysteretic magnetization loops, expanding on the measurement principles presented by Klingbeil et al.^6,7^. A unique set of gain and delay invariant material-specific scalars for any combination of temperature and magnetic field is extracted from the loops, enabling parallel measurement of both quantities.

Known techniques of magnetic field sensing include fluxgate sensors^8^, Hall sensors^9^, magnetoresistive sensors^10^, fiber-optic optoelectronic sensors^2^, magnetoelectric sensors^11^, optically pumped magnetometers^12^, superconducting quantum interference devices (SQUID)^13^, and nitrogen-vacancy defect magnetometry^14^. For temperature sensing, magnetic approaches based on changes in susceptibility^6^ or saturation magnetization^15^ have been proposed. Optical methods include infrared^16^, luminescence^17^, fiberoptic sensors^18^, and thermochromic thermography^3^. More established thermal sensors are thermistors, thermocouples, resistance temperature detectors, and semiconductor devices^19^. Examples for combined thermal and magnetic sensing are based on fiber optics^20^ or as imaging using magneto-optical microscopy^3,21,22^.

In general, magnetization processes during magnetic field application can be governed by magnetic domain wall motion, domain nucleation and annihilation, as well as magnetization rotation. These processes result from competing exchange, anisotropy, magnetoelastic and magnetostatic energies, together with the dynamics of the domain pattern^23^. Any physical quantity that modifies these energy contributions, for example by changing the exchange stiffness, anisotropy constant, or saturation magnetization, will consequently affect the magnetization loop shape. These closed magnetic hysteresis loops as a signal over time can be adequately represented by their harmonic components^24,25^. A limited set of harmonic amplitudes and phases is sufficient to describe the essential features of the loop shapes^24,26^, which can be experimentally determined with high sensitivity via lock-in detection^27^. Measurement schemes using individual harmonic amplitudes^28^ and phases^29^ of a magnetization signal have been demonstrated. In ref^24^., special attention is given to the temperature dependence of harmonic amplitudes and phases. Correlations of the harmonic components to relevant magnetic material properties is possible^30^. In experimental implementations, however, the measured harmonic amplitudes are affected by experimental scaling factors, making multiparametric sensing and calibration challenging, where a robust and decoupled measurement approach is highly desirable^2^. We therefore express the signal through ratios of harmonic amplitudes, providing repeatable, shape-specific parameters that are unaffected by experimental scaling, amplitude or calibration conditions. Such strategies, utilizing magneto-optical (MO) signals using normalized amplitudes, have been demonstrated to extract magnetic vector components^31^ or temperatures^7^. Similarly, by considering phase differences between harmonics, a normalization equivalent to the amplitude relation is achieved. Here, we utilize both phase and amplitude relations to form a set of sample-specific shape parameters acting as a fingerprint suitable for combinatorial measurement of physical quantities.

In this work, we focus on temperature and a single static magnetic field component, measured via MO readout of magnetization response, providing rapid contactless measurement with inherent galvanic isolation. As a sensor, we use an MO indicator film with out-of-plane magnetic anisotropy^6,15,21,22,32,33^. The active layer is a transparent ferrimagnetic bismuth-substituted yttrium iron garnet (Bi:YIG) film. It exhibits a large Verdet constant *V*, which quantifies the strength of the Faraday effect and results in a high MO signal-to-noise ratio^34^. In transmission, the Faraday effect rotates the polarization axis of light proportional to the magnetization *M* along the propagation direction *k*. For reflection-mode operation, a mirror is placed at the backside of the film, so that the light passes the indicator layer twice, effectively doubling the MO rotation. The resulting Faraday rotation *β*_MO_ for perpendicular incidence of light is given by^35^

1 $${\beta}_{\mathrm{M}\mathrm{O}}\left(T,H,t\right)\approx V\left(T\right)\cdot2d\cdot\left(\overrightarrow{M}\left(T,H,t\right)\cdot\overrightarrow{k}\right)$$

where *d* is the film thickness. The rotation depends on the magnetic field *H*, the temperature *T* and time *t* due to the corresponding response of the magnetization. With subsequent polarization optics, this can be converted into an intensity change detectable by photodiodes.

## Results

### Magneto-optical loops

**Fig. 1:**
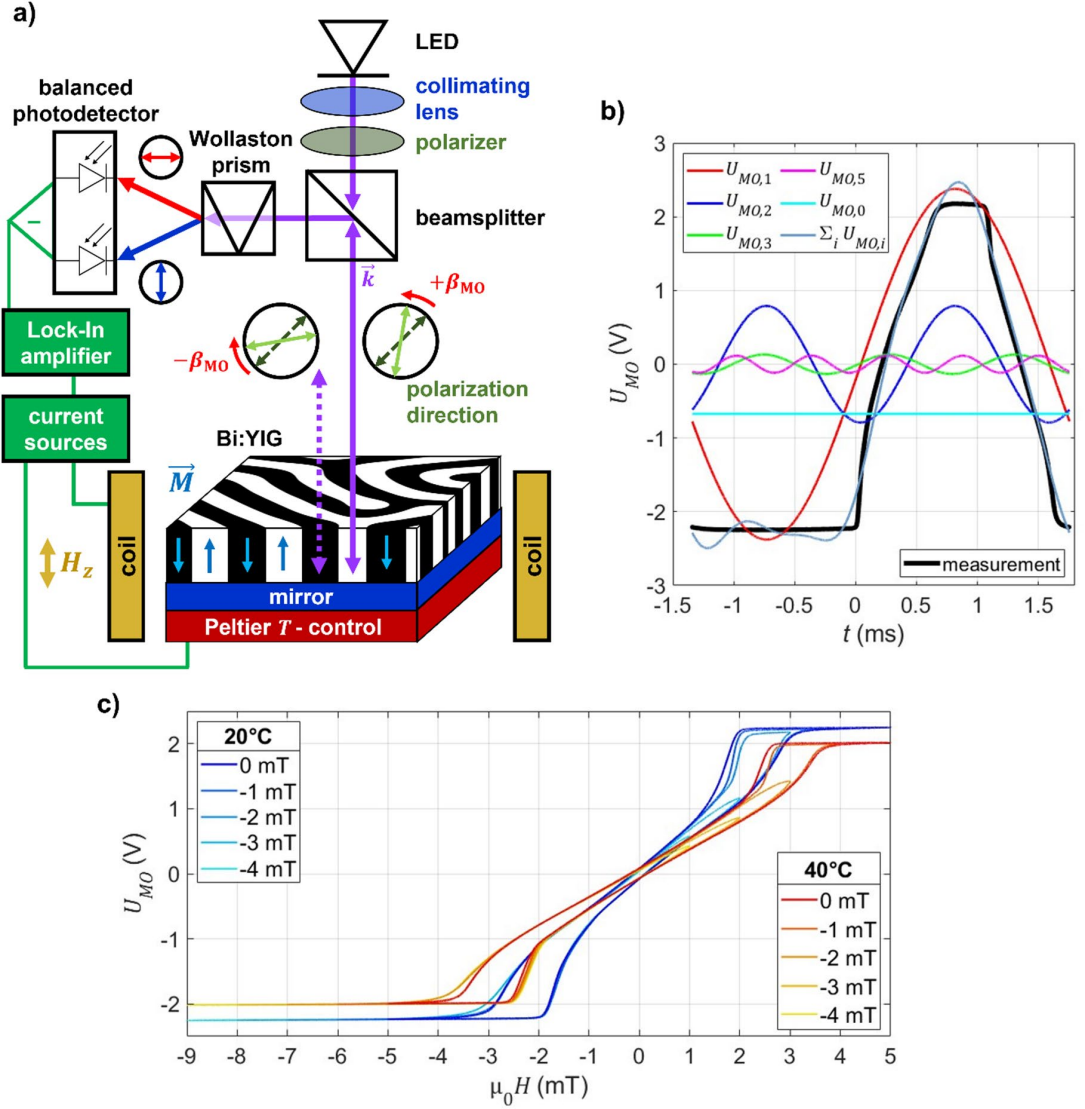
Experimental fundamentals. **a**) Schematic magneto-optical dual quadrature setup. The lock-in amplifier output drives a current source producing an alternating perpendicular magnetic field µ_0_*H* via an electromagnet, altering the magnetization *M* of a Bi:YIG film. This causes a polarization rotation *β*_MO_ of incoming linearly polarized light at perpendicular incidence. Then, the reflected light beam is split into two orthogonally polarized beams by a Wollaston prism. The resulting intensity difference is detected with a balanced photodetector and fed into the lock-in amplifier. The temperature *T* is actively controlled using a Peltier-element. **b**) MO signal over time *t* at 20 °C and µ_0_*H*_off_ = − 2 mT, decomposed into its *i*^th^ (1st, 2nd, 3rd, and 5th ) harmonics *U*_MO, i_, as well as the average signal offset *U*_MO,0._ Their sum S_i_ *U*_*MO*,i_ closely approximates the measured real space MO signal. **c**) Averaged magneto-optical (MO) signal *U*_MO_ loops to applied alternating magnetic excitation field of µ_0_*H*_AC_ = 5 mT at 323 Hz for different temperatures and additional bias magnetic fields µ_0_*H*_bias_.

MO magnetization loops are recorded using an MO dual-quadrature setup^36^, shown schematically in Fig. 1a and described in detail in the methods section. The averaged MO signal from the balanced photodetector output *U*_MO_(*H*) obtained from the Bi:YIG film excited by an alternating sinusoidal magnetic field µ_0_*H*_AC_ = 5 mT is represented in Fig. 1b. The observed loop shapes are characteristic of thin films with perpendicular anisotropy^23^, which typically show a corresponding maze domain pattern in the remanent state, as seen in Fig. 1a. (See also^6,7^ for details).

For low applied magnetic fields, the sample response is approximately linear, as magnetic domain wall movement alters the relative domain width. For larger fields, the differential MO response increases as domain nucleation and annihilation effects start to dominate^37^. Within the investigated temperature range between 13 °C and 66 °C, the susceptibility near zero field, the saturation magnetization, and the hysteresis loss decrease with temperature, while the nucleation and annihilation fields increase.

In Fig. 1b an exemplary loop measured at 20 °C and bias field of µ_0_*H*_bias_ = ‒2 mT is represented as a time-domain signal over one excitation period. The results of a Fourier decomposition of this signal into the respective harmonic components *U*_MO,*i*_ consisting of harmonic amplitude *A*_MO,*i*_ and phase *ϕ*_MO,*i*_ are displayed as well. The first (fundamental), second, third and fifth harmonic, as well as the average signal offset *U*_MO,0_, are shown. Their sum closely reproduces the measured signal, demonstrating that these four harmonics capture the essential features of the hysteresis loop except the signal offset. Higher harmonics are not necessary to uniquely quantify temperature and magnetic fields. They are omitted in the following analysis. Nonetheless, all described concepts also work for higher harmonics.

The influence of magnetic bias fields µ_0_*H*_bias_ on the *U*_MO_(*H*)-loop shapes is shown in Fig. 1c. At small bias fields, the loop shape changes only slightly in the first quadrant, since pinned, not previously annihilated domains facilitate domain-wall propagation. For larger bias fields, annihilation for positive fields is suppressed. In the third quadrant, the gap between nucleation and annihilation field widens. As the sinusoidal excitation is shifted by increasing bias fields, the time derivative of the applied field increases in the third quadrant. Due to magnetic viscosity in the switching behavior of perpendicular domains, the magnetization response is delayed with respect to the applied field. Similar processes are observed in OOP anisotropy thin films^38^.

### Interpretation of harmonic coefficients

The physical interpretation of individual harmonic amplitudes and phases in a signal over time, specifically in terms of the underlying magnetization loop, is described next. To establish a clear framework for interpreting the relationship between harmonic coefficients and characteristic loop features, a series of exemplary magnetization loops is calculated.

The MO signal *U*_MO_ described via its Fourier components comprises of the sum of harmonic components *U*_MO,*i*_ (*t*) = *A*_*i*_sin(*i* ∙ 2π *f t* + *ϕ*_*i*_) at the harmonic *i*, time *t* and frequency *f* based on the harmonic amplitude *A*_*i*_ and phase *ϕ*_*i*_. Mapping the time to a corresponding sinusoidal normalized applied field *H*/*H*_AC_ = sin(2π *f t*), the signal can be described as a function of magnetic field using the harmonic components:

2 $${U}_{MO}\left(H\right)=\sum_{i}{A}_{i}\mathrm{sin}\left({i\cdot\mathrm{sin}}^{-1}\left(\pm H/{H}_{AC}\right)+{{\upvarphi}}_{i}+\frac{\pi}{2}\mp\frac{\pi}{2}\right)$$

**Fig. 2:**
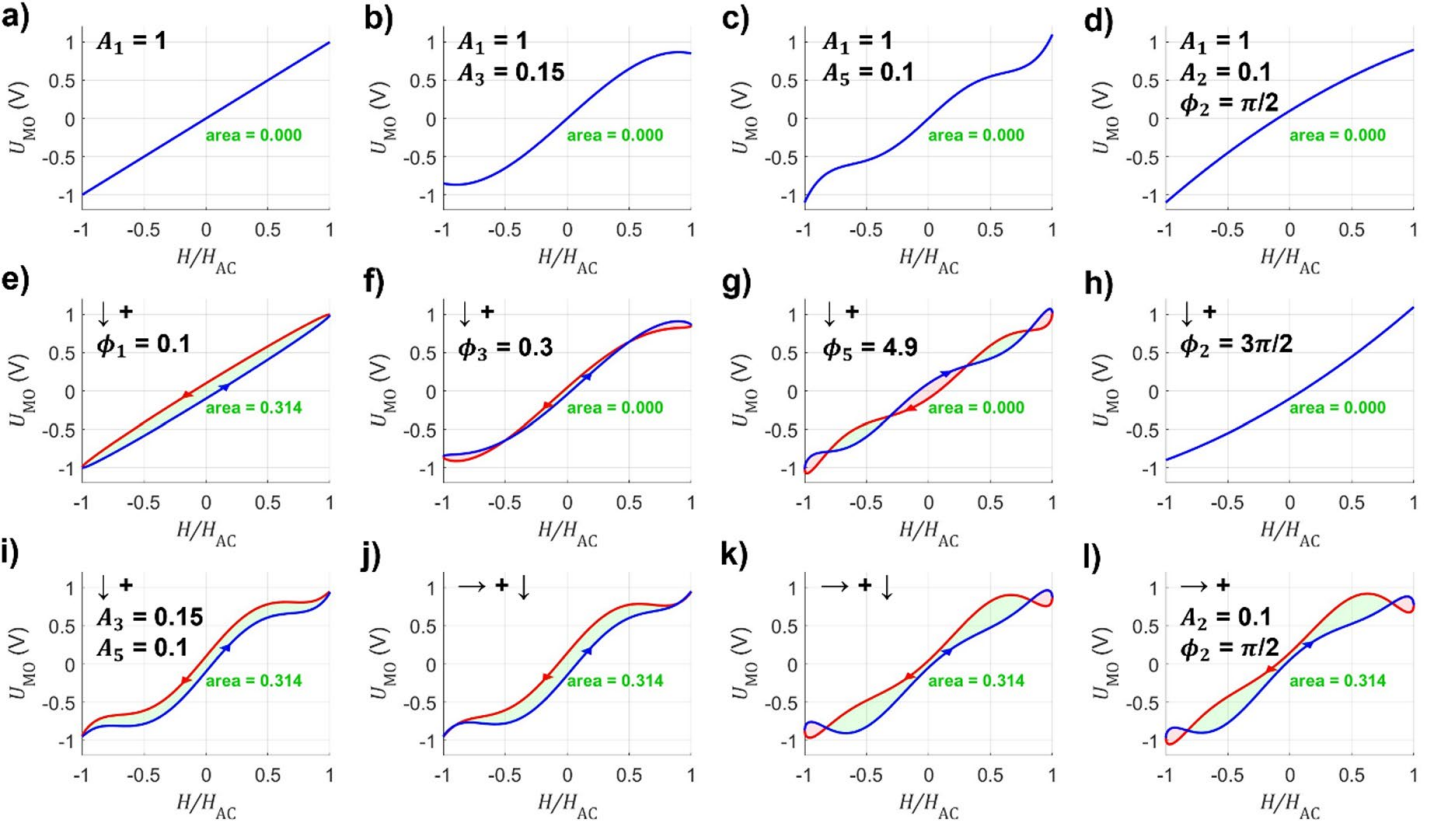
Visualization of magnetization loops for varying harmonic signal amplitude and phase. **a**) Anhysteretic linear response of the fundamental harmonic amplitude, acting as the baseline for all graphs. **b**) to **d**) show the loops with the addition of a single other harmonic *i* with amplitude *A*_*i*_ at phase zero, b) *A*_3_, c) *A*_5_ and d) *A*_2_. The loops **e**) to **h**) are the same as above, but a single additional phase *ϕ*_*i*_ is introduced, e) *ϕ*_1_, (f) *ϕ*_3_, (g) *ϕ*_5_ and (h) *ϕ*_2_. The interim results (i) and (j) represent a step wise construction of the exemplary loop consisting of three harmonic components from the results above, which is finally reached in **k**). The addition of the second harmonic to this loop for a non-point-symmetric case is indicated in **l**). The abscissa is the normalized applied field *H/H*_AC_ during the period of an alternating magnetic field. The signal amplitude *U*_MO_ is set arbitrarily. The blue line represents the ascending branch of the hysteretic magneto-optical signal, the red line indicates the descending branch.

The two resulting solutions represent the ascending or descending branch of the magnetic hysteresis. Each harmonic component is examined individually and subsequently combined, serving as a visual guide for the interpretation used in the following sections. The results are presented in Fig. 2.

The fundamental harmonic corresponds to the purely linear, anhysteretic response (Fig. 2a). Higher odd harmonics introduce nonlinearities while maintaining point symmetry. The third harmonic (Fig. 2b) yields a three-segment gradient (low–high–low), while the fifth harmonic (Fig. 2c) further refines the loop with a five-segment pattern without altering the average. In contrast, even harmonics break the point symmetry and shift the average response (Fig. 2d). Their contribution corresponds to loop asymmetry and is thus essential for measuring magnetic fields in this example. The amplitude of the second harmonic indicates the magnitude of the asymmetry, while its phase defines its direction (Fig. 2h).

The phase *ϕ*_1_ of *U*_MO,1_ primarily reflects the hysteresis area of the loop. It indicates the extent to which magnetization lags behind the applied field, thus directly setting the loop opening area together with its amplitude (Fig. 2e). Phases of the higher odd harmonics do not alter the total loop area but redistribute it across different field regions (Fig. 2f and g). A phase shift introduces alternating positive and negative areas equal to the harmonic order, effectively localizing the hysteretic contributions. In the shown example, the *ϕ*_3_ emphasizes the area difference between the center and the saturation regime at the edges, while *ϕ*_5_ captures the increased area for intermediate applied fields of the nucleation regime in relation to the reduced area around zero field. Additionally, a phase change of π reverses the influence of any harmonic amplitude (Fig. 2h), and thus also the sign of the areas.

Combining the complete first harmonic with the higher odd amplitudes yields loop shapes that resemble experimental measurement data. As shown in Fig. 2i, the inclusion of the odd harmonic amplitudes broadens the plateau at high fields, capturing the onset of saturation. Adding the higher odd phases (Fig. 2j and k) redistributes the hysteresis area, thus reproducing the characteristic features of the measured loops, where most of the hysteretic area originates from the intermediate field regime. Since each harmonic amplitude and phase encodes a distinct structural aspect of the loop, their combination provides a sufficiently diverse and complementary set of descriptors to serve as the foundation for multiparametric sensing.

### Shape parameters

The characteristic shape parameters can be differentiated into amplitude *P*_*A*_, phase *P*_*ϕ*_ and combined parameters *P*_*Aϕ*_. The amplitude parameters are divisions between two harmonic amplitudes. This normalization cancels out the excitation amplitude^7^. As the signal to noise ratio decreases with higher harmonics, the first harmonic is always used as a reference:

3 $${P}_{A,i}={A}_{i}/{A}_{1}$$

Note that this relation leads to the cancellation of the saturation signal, and thus results in information loss.

For the harmonic phases, the initial delay must cancel out. This requires the phase parameters to be the difference of a higher harmonic phase to the fundamental. However, the *ϕ*_1_ needs to be scaled accordingly, as it is the harmonic number *i* times less susceptible to external phase changes, because its period is correspondingly larger:

4 $${P}_{\varphi,i}=\mathrm{s}\mathrm{i}\mathrm{n}\left({\varphi}_{i}-i{\varphi}_{1}\right)$$

Additionally, the sine is calculated to prevent ambiguities related to the multiples of *π*. A combined parameter *P*_*Aϕ,i*_ is a multiplication of the amplitude and phase parameter.

### Excitation amplitude

**Fig. 3:**
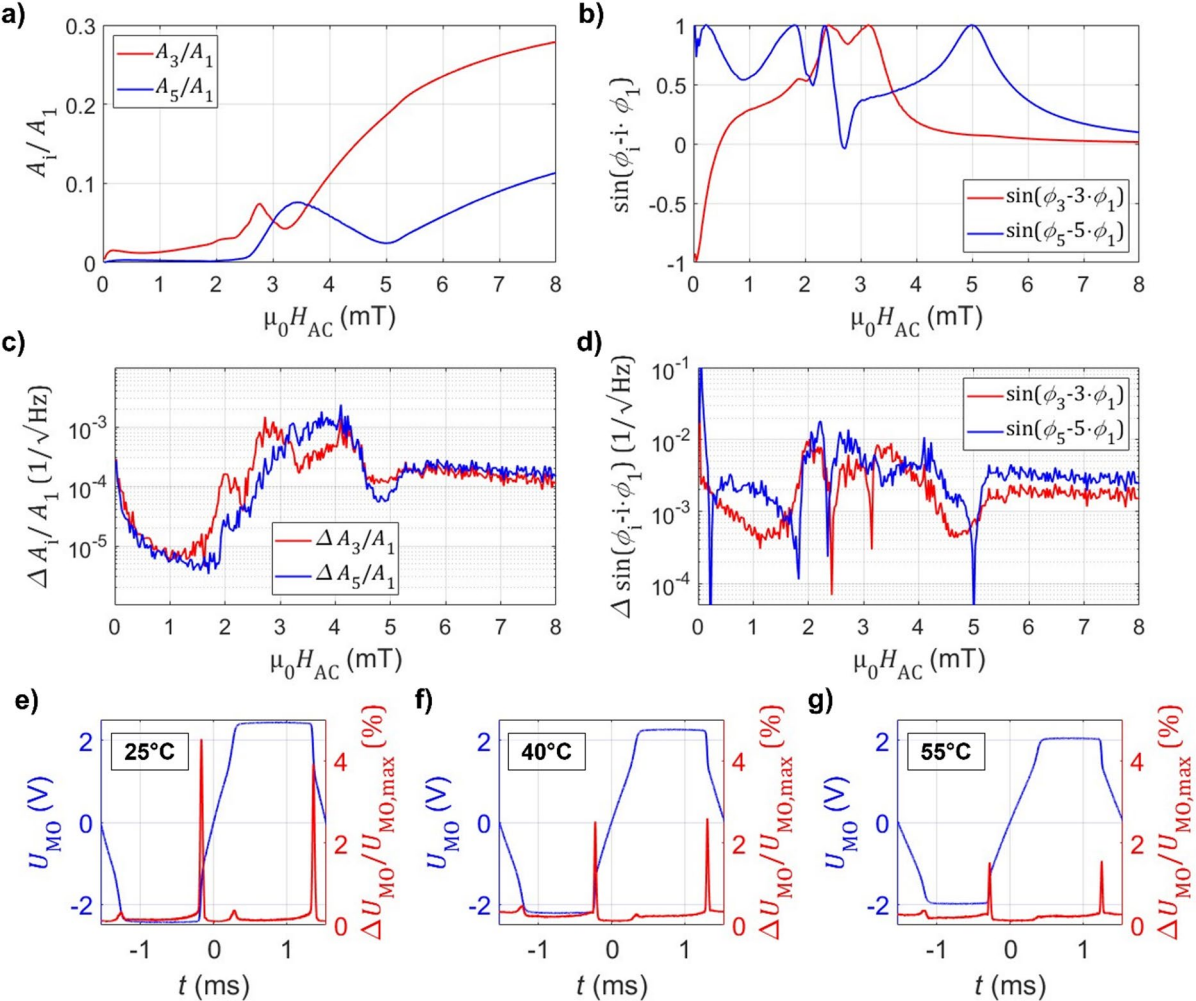
Influence of the excitation amplitude and noise characteristics. **a**) Normalized harmonic amplitudes *A*_i_/*A*_1_ as a function of the applied field amplitude µ_0_*H*_AC_ and **b**) characteristic phase difference parameter sin(*ϕ*_i_-i*ϕ*_1_). Respective noise densities for **c**) amplitude parameter and **d**) phase parameter. Measurements are performed with an excitation frequency of 323 Hz at 25 °C and no bias field. **e**) to **g**) Normalized root mean square deviation ∆*U*_MO_/*U*_MO, max_ between individual hysteresis loops for each point in time during the period at µ_0_*H*_AC_ = 5 mT at e) 25 °C, (f) 40 °C and (g) 55 °C.

The Fourier components of the magnetization loop depend on the excitation amplitude *H*_AC_. The measured behaviors of harmonic shape parameters at 25 °C are presented in Fig. 3a and b. For small excitations, the loop is primarily linear and the higher harmonics are small. The negative *P*_*ϕ*,3_ indicates an increased gradient at the edges of the loop. With increasing *H*_AC_, nonlinearities become more pronounced and the harmonic amplitudes grow. Several extrema appear within the magnetic field range between nucleation, annihilation and saturation field. Afterwards it stabilizes, as the time signal starts to converge to a rectangular shape (1/3 for *P*_*A*,3_ and 1/5 for *P*_*A*,5_, and both phase parameters to zero). As the measurement is performed without a magnetic bias field and thus asymmetry, the second harmonic is neglected.

In addition to modifying the magnetization loop below the saturation field, increasing the excitation amplitude changes the detected loop shape when plotted to the normalized applied field *H/H*_AC_. As shown in Fig. 2., this leads to dependencies of the Fourier components to excitation field also above the saturation field, as more time is spent during saturation.

The corresponding noise of the shape parameters is plotted in Fig. 3c and d. At low magnetic bias fields, the noise remains small because domain wall motion is largely repeatable between successive loops. The noise rises sharply in the field range between the onset of nucleation and complete saturation. This originates from magnetic jitter in the loops due to variations in magnetic domain nucleation, and to some minor degree domain annihilation, as these processes are inherently stochastic. This becomes visible in the peaks in the deviation between different individual magnetization loops in Fig. 3e and g at the position of the nucleation regime. At µ_0_*H*_AC_ ≈ 4.5 mT, the noise level decreases again, as (nearly) complete magnetic saturation is reached, reducing the uncertainty regarding nucleation sites. Additionally, as the excitation is sinusoidal, the standing times within the nucleation regime decrease. Still, the magnetic noise from uncertainty in the nucleation is the limiting factor regarding the measurement accuracy. This uncertainty decreases at higher temperatures, as visible in the decrease of the peak height in Fig. 3e-g with temperature. For all subsequent measurements, the excitation field is set to µ_0_*H*_AC_ = 5 mT, as it provides pronounced nonlinear behavior, while avoiding the high-uncertainty regime near nucleation and remaining below the field strength where the loops lock into a dominantly square waveform.

The influence of the frequency on the shape parameters is minor compared to the excitation amplitude. Lower frequencies increase the contribution of 1/*f* noise, whereas higher frequencies are limited by the inductive impedance of the solenoid and electrical resonance in the drive circuit. A frequency of *f* = 323 Hz is selected as a compromise between these constraints while avoiding harmonics of the mains frequency.

### Parameter map

**Fig. 4:**
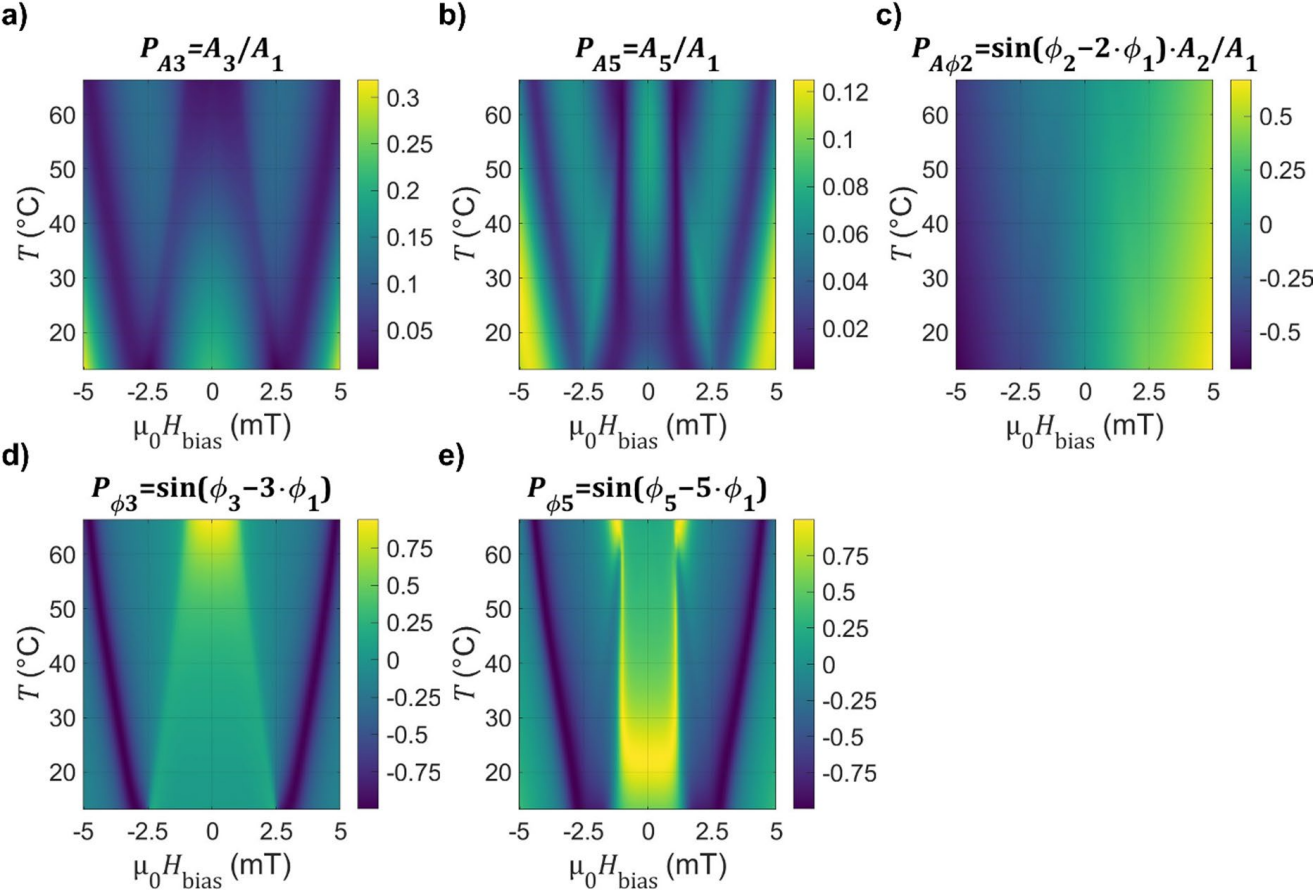
Characteristic parameter map. Normalized harmonic amplitude parameter maps as a function of temperature *T* and applied magnetic offset field µ_0_*H*_bias_ for **a**) the third harmonic *P*_*A*,3_ and **b**) the fifth harmonic *P*_*A*,5_. **c**) Combined harmonic amplitude and phase parameter map based on the second harmonic *P*_*Aϕ*,2_ exhibiting a continuous behavior to the magnetic bias field. Measured characteristic harmonic phase parameter maps to temperature and applied magnetic bias field of **d**) the third harmonic *P*_*ϕ*,3_ and **e**) the fifth harmonic *P*_*ϕ*,5_.

The shape parameters are systematically measured twice for varying temperatures and magnetic fields. The resulting maps (Fig. 4) reveal a complex and nonlinear behavior.

The uneven harmonic amplitude parameters *P*_*A*,3_ and *P*_*A*,5_ (Fig. 4a and b) are magnetic field symmetric and show an alternating pattern with magnetic field bias forming valleys and ridges. The height of the ridges decreases with rising temperature, as the loops become more linear with the increase of the saturation field and decrease of hysteresis. Also, the positions of the resulting valleys shift with temperature. These positions in the amplitude maps also coincide with rapid phase changes as illustrated in Fig. 4d and e. For the 3rd harmonic (Fig. 4a and d with ${P}_{\mathrm{A},3}$ and ${P}_{{\upvarphi},3}$), the outer valley corresponds closely with the annihilation field. Most of the dependence of the valleys to temperature can be connected to the shift of nucleation and annihilation field with the corresponding movement of the hysteretic region.

For the fifth harmonic (Fig. 4b and e with *P*_*A*,5_ and *P*_*ϕ*,5_) between bias fields of µ_0_*H*_bias_ = − 1 and + 1 mT, a rectangular region can be identified. Within this region, the harmonic components are governed primarily by temperature, as the bias field is still smaller than the smallest area cell (see Fig. 2g). Beyond that, it shows the temperature dependent valleys and ridges.

The combined parameter using the second harmonic *P*_*Aϕ*,2_ is a continuous function with magnetic field. The contribution of the phase is essentially either − 1 or + 1, thus indicating the direction of the bias field. The amplitude relation scales approximately linear to the value of the bias field, with a temperature dependent gradient to the bias field.

Both independent measurements generate equivalent results. The parameter maps are independent on magnetic or thermal history, as saturation is reached in at least one quadrant of the magnetization loop during each cycle.

### Demonstration

The sensor calibration is based on the parameter maps shown in Fig. 4. Two different solving schemes are implemented: (i) a lookup table (LUT) approach using a multidimensional minimization. (ii) a random forest regressor (RFR) machine learning model (see Methods section for details).

**Fig. 5:**
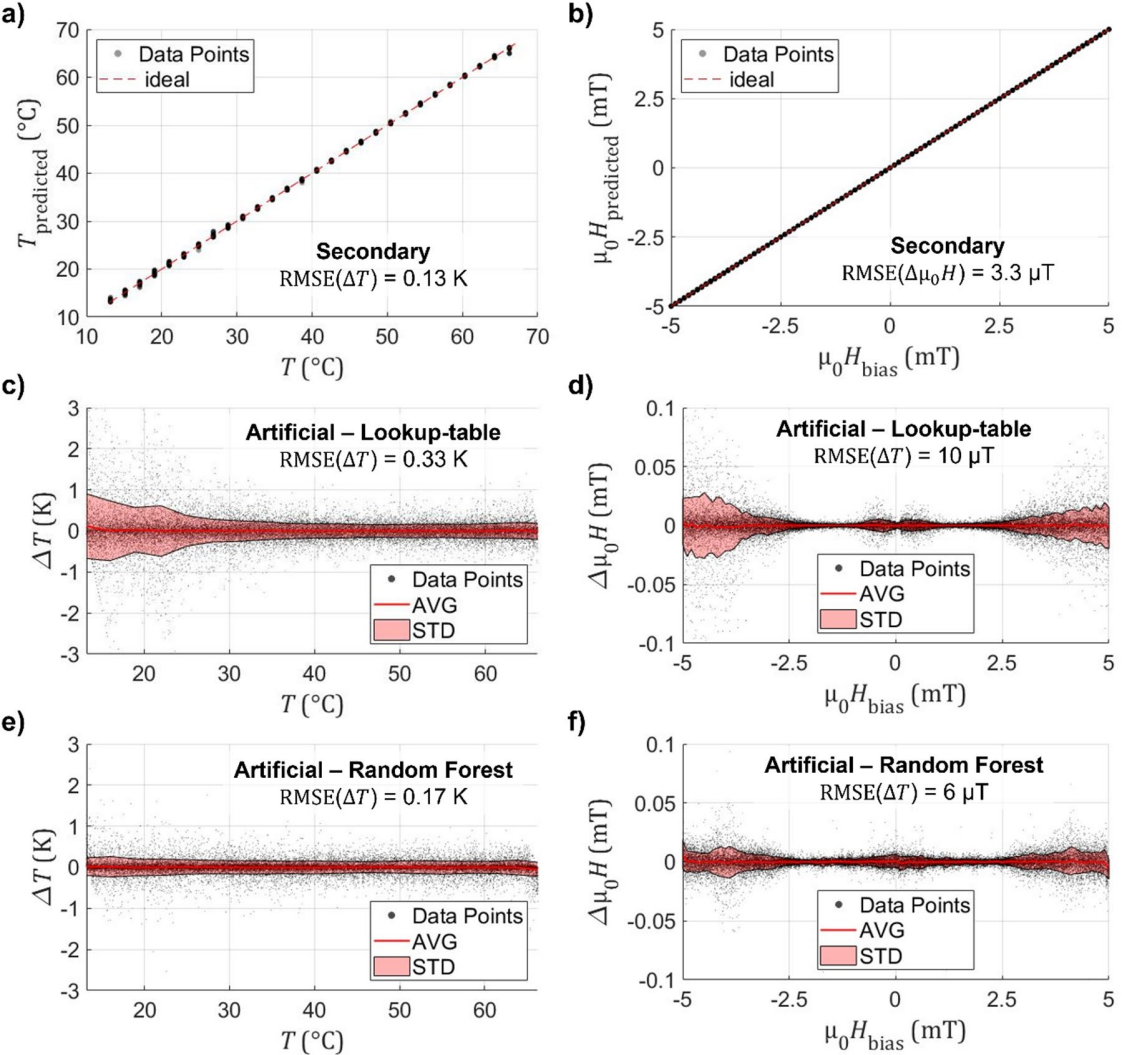
Measurement scheme accuracy. **a**) Predicted to applied temperature *T* for all applied magnetic bias fields µ_0_*H*_bias_ of a secondary independent measurement using a lookup table calibration. **b**) Predicted to applied magnetic field for all temperatures of the same secondary measurement. As the secondary measurement is highly averaged, a) and b) consist primarily of systematic measurement inaccuracies. **c**), **d**) Residual results of the lookup-table solver on a simulated dataset created based on the secondary measurement with added 1/√Hz noise to all harmonic components for c) temperature and d) magnetic field. Residual results for **e**) temperature and **f**) magnetic field determined with the random forest regressor based on the same artificial dataset.

To evaluate repeatability and systematic accuracy, an additional, independent validation measurement is performed. The parameters are extracted and the results of the solvers are compared to the applied field and temperatures, illustrated in Fig. 5a and b. Though only the lookup-table result is shown, both solvers produce equivalent results. The predictions coincide well with the reference measurement and have an average root mean square deviation of approximately ∆*T*_sys_ = 0.13 K and ∆µ_0_*H*_sys_ = 5 µT, which indicate the systematic error. This contains possible drift of the excitation amplitude and frequency between the measurements, imperfections of the interpolation fit used as calibration, as well as actual temperature variations between the thermocouple reference and the sensor. Based on the gradients of *P*_*A*,3_ to the excitation amplitude (Fig. 3a) and to temperature (Fig. 4a), the order of magnitude of the temperature variation caused by drift in the excitation amplitude (at room temperature and *H*_bias_ = 0) can be estimated as ∆*T*_*H*AC_ ≈ 12 K/mT or 0.6 K for 1% drift. For the magnetic field, the systematic error also includes possible variations regarding urban magnetic noise.

A statistical analysis of the measurement noise is realized by creating an artificial dataset based on the secondary measurement. This artificial dataset contains data points within the whole measurement range. The respective interpolated parameters are determined from a lookup table, but additional 1/√Hz-noise as measured is added randomly to all artificial datapoints. The results of the lookup-table solver are shown in Fig. 5c and d. It exhibits a ∆T_LUT_ = 0.33 K and µ_0_∆*H*_LUT_ = 10 µT. The machine learning results are shown in Fig. 5e and f with a lower error of ∆T_RFR_ = 0.17 K and µ_0_∆*H*_RFR_ = 6 µT. In addition to the noise contributions mentioned above, this also contains the numerically determined inaccuracy. Nonetheless, the achievable residual might be lower, as the different Fourier components are not linear independent from each other.

A higher standard deviation of the temperature residual is obtained for lower temperatures. This coincides with the observation of higher magnetic jitter in the domain nucleation regime in the magnetization loop (see Fig. 3e-g) and thus signal noise. For the magnetic bias field, the results are symmetric with applied field. For low fields, the standard deviation decreases with applied field. This originates from the comparatively low gradients for all uneven harmonic parameters to applied field. A minimum is reached, as the nucleation jitter on one side of the hysteresis loop decreases. This effect roughly corresponds to a lower excitation as depicted in Fig. 3c. For very high applied fields, the total amount of time outside of saturation decreases, thus decreasing the overall signal to noise ratio of the detectable signal.

Overall, the average deviation is very close to zero. Only near the edges of the measurement range does it deviate slightly, because the distributions become asymmetric close to these edges as solutions beyond the range are not taken into account for in either solving mechanisms.

The lower average residual achieved by the machine learning model results from the training of the random forest regressor on noisy data, enabling generalization for deviations as well. Since the noise distribution of the Fourier components is inhomogeneous, machine learning learns to weight parameters according to their local reliability. Consequently, the random forest regressor has fewer outliers and outperforms the iterative lookup-table solver in terms of accuracy.

The random forest regressor requires an extensive training on a sufficiently large and homogenous dataset to ensure adequate generalization and to prevent overfitting. However, the model only has to be trained once, as the shape parameters are robust. Due to the non-linear multidimensional optimization problem, datapoint inversion using a random forest regressor is computationally significantly less expensive than using the iterative look-up-table solver. This discrepancy in the computational cost grows with increasing numbers of measurement inputs and outputs. As machine learning can be easier extended for more measurement parameters than a lookup table, it provides a sensible alternative for higher-dimensional nonlinear sensor readout.

Different, simpler solving algorithms using less parameters are generally possible as well. In this example, at least three parameters would be necessary, as the shape parameters are not steadily rising functions, evidenced by the ridges in the parameter maps. In combination with measurement noise, this increases the probability of outliers and leads to decreased maximal achievable precision.

## Conclusion

A magneto-optical method for parallel temperature and magnetic field sensing is proposed, which utilizes material characteristic shape parameters of the magnetization loop constructed from relations of the Fourier components detected at harmonics of a magnetic excitation. The magnetization measurement has been realized with a MO readout of a perpendicular Bi:YIG MO indicator film sensor in a dual quadrature setup. The Fourier components are detected directly via a lock-in amplifier, and their interpretation with regard to the shape of the magnetization loop is discussed. The dependence of the shape parameters of temperatures and magnetic field is investigated and can yield a mean equivalent noise density of 0.17 K/√Hz and 6 µT/√Hz using a random forest regressor machine learning algorithm, corresponding to 0.3% and 0.1% of the measurement range respectively. The limiting factor in terms of noise is the magnetic nucleation variance of the magnetic sensor film. Since the shape parameters used are invariant in terms of gain and delay, the presented method is robust and therefore does not require recalibration.

The MO approach allows for a possible integration with fiber optics^39^, and provides galvanic isolation with rapid readout. Nonetheless, the presented analysis method can be applied to other sensing techniques, such as inductive readout, or even other excitable materials, such as ferroelectrics^40^, as well. The prerequisite is an excitable workpiece, yielding a non-linear response which is dependent on physical quantities. Thus, the amount of possibly measurable characteristics with the presented MO scheme is extendable as well, for example adding more magnetic field components. While requiring a higher dimensional initial calibration, this process could be facilitated by utilizing machine learning.

## Methods

### Sensor material

The used MO sensor is a bismuth substituted yttrium iron garnet epitaxially grown on a < 111 > gallium gadolinium garnet with growth induced perpendicular anisotropy. The ferrimagnetic film is approximately 3 μm thick and exhibits a Néel temperature of 138 °C. A mirror and protection layer are situated on top of the magnetic film. The maximal MO polarization rotation after reflection in out-of-plane saturation is *β*_MO, sat_ = 7.45° at 21 °C around a wavelength of 554 nm. It is a commercially available sensor “Type A” from matesy GmbH^41^.

### Dual-quadrature optical setup

A green LED light source (554 nm) is fed into a multimode fiber. Its output is then collimated into a polarizer. The polarization axis is oriented at a 45° angle to the subsequent polarization-maintaining beam splitter. After transmission, the light beam falls perpendicular on the sensor and is reflected at a back mirror, resulting in a MO Faraday rotation. Afterwards, the light is reflected at the beam splitter into a lens and a Wollaston prism. Here, it is separated into two orthogonally polarized beams. Due to the 45° angle of the polarizer to the Wollaston prism, the intensities of both beams are on average equal. The lens focusses these beams onto a balanced photo diode. The resulting signal is low noise in regards to changes of the overall light intensity and is used as the primary MO signal. All displayed magnetization loops are averaged over 100 periods of the excitation field at the respective conditions of magnetic bias and temperature.

### Magnetic field control

The magnetic field excitation is created via a current in a solenoid driven by a four-quadrant voltage-controlled power source in current mode. The solenoid has an outer diameter of 80 mm, an inner diameter of 40 mm, a height of 22 mm, with a wire diameter of 1.32 mm and a magnetic field calibration of 4.17 mT/A. The voltage input is connected to the primary output of the lock-in amplifier. The resulting magnetic field is oriented perpendicular to the sensor film and is calibrated for varying frequencies. An additional direct current offset is added to create the magnetic bias field. All shown measurements of the harmonic components are carried out with a 5 mT sinusoidal alternating magnetic field at 323 Hz.

### Temperature control

All measurements are carried out with active temperature control, with sufficient time between temperature steps to allow the system to reach a stable state. The sensor is thermally coupled to a Peltier element via a vertical stack of thin (1 mm) copper plates to minimize parasitic effects from currents in the Peltier element and provide thermal mass, while the plating reduces the occurrence of eddy currents at higher frequencies. A type K thermocouple placed 2 mm below the sensor within the copper plating constantly measures the temperature and acts as a reference. The backside of the Peltier element is thermally stabilized with a water-cooling system. An active PID control via a secondary current source is implemented. The temperature range between 13 °C and 66 °C is investigated.

### Lookup table interpolation solver

For every parameter *P*_i_, a lookup table with regard to *T* and *H*_bias_ is created based on the measurement in Fig. 4. Cubic interpolation is applied for parameters using harmonic amplitudes and linear interpolation is applied for the phase parameters. The optimization function *O*(*T*,* H*_bias_) is the sum of the normalized squares of the differences between the actual measured values and the interpolated values from the calibration lookup Table

5 $$O\left(T,{H}_{\mathrm{b}\mathrm{i}\mathrm{a}\mathrm{s}}\right)=\sum_{i}{\left(\frac{{P}_{\mathrm{i},\mathrm{m}\mathrm{e}\mathrm{a}\mathrm{s}}-{P}_{\mathrm{i},\mathrm{l}\mathrm{o}\mathrm{o}\mathrm{k}\mathrm{u}\mathrm{p}}\left(T,{H}_{\mathrm{b}\mathrm{i}\mathrm{a}\mathrm{s}}\right)}{{P}_{\mathrm{i},\mathrm{m}\mathrm{a}\mathrm{x}}-{P}_{\mathrm{i},\mathrm{m}\mathrm{i}\mathrm{n}}}\right)}^{2}$$

This is minimized numerically using an iterative grid refinement with regard to *T* and *H*_bias_ for any measured datapoint. This allows for robust solving despite the large number of local minima present in the optimization function for the presented data. The output is limited to the measurement range.

### Random forest regressor

A random forest regressor^42^ is employed to predict temperature and magnetic field from the harmonic parameters. For training, an artificial dataset with added noise has been created from the calibration measurement. This enables the creation of a large number of datapoints randomly distributed within the experimental range, thus utilizing the strong law of large numbers to prevent overfitting^42^. The random forest consists of 96 estimators with a set minimum of 3 branches. The resulting average tree depth of the model is 37 with 1.14 × 10^6^ artificial datapoints used. After training, the model was validated using an independent test dataset derived directly from the original calibration measurement.

## Supplementary Information

Below is the link to the electronic supplementary material.

Supplementary Material 1

## Data Availability

The datasets generated and analyzed during the current study are available from the corresponding author on reasonable request.
