# Supplementary material for: Multiparametric robust sensing via readout of characteristic magnetization loops
Source: Sci Rep. 2026 Mar 3;16:8148. doi: 10.1038/s41598-026-42763-x (PMC12960790; doi:10.1038/s41598-026-42763-x)
Supplement: Supplementary file 1 — Supplementary Material 1 [file 41598_2026_42763_MOESM1_ESM.pdf]

Multiparametric robust sensing via readout of characteristic magnetization loops

Michael P. Path[1]*, Michael Vogel[1,2], Jeffrey McCord[1,2]

[1]Nanoscale Magnetic Materials - Magnetic Domains, Department of Materials Science, Faculty of Engineering, Kiel University, 24143, Kiel, Germany

[2]Kiel Nano, Surface and Interface Science (KiNSIS), Kiel University, 24118 Kiel, Germany

*Corresponding Author: mipa@tf.uni-kiel.de

Supplement

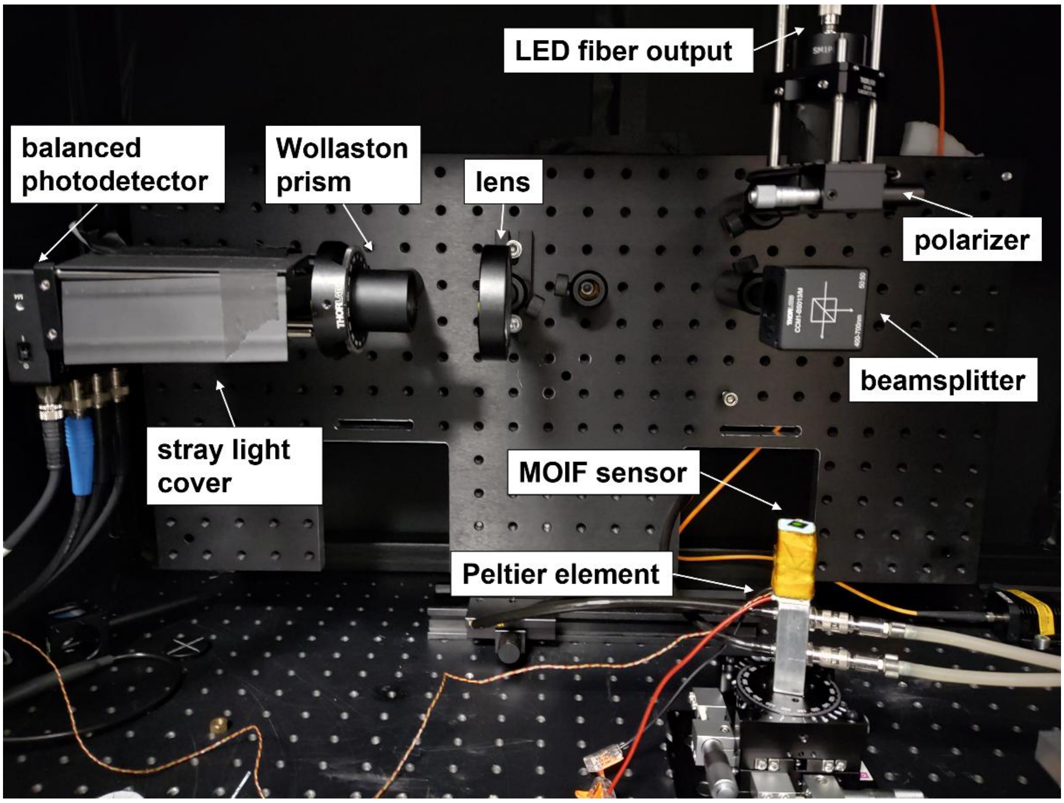

Figure S1. Photograph of the experimental setup analogous to Figure 1a.

Details of Demonstration Measurement and Calibration Time

Both individual measurements of the parameter maps were recorded on the same day, each taking approximately 4 hours. All subsequent calculations are performed on the same workstation with a XEON CPU E5-1630 v4 @ 3.7 GHz and 32 GB RAM.

The generation of the artificial training dataset require ~2 hours. The training of the random forest regressor machine learning model requires ~47 minutes. Inferring temperature and magnetic field from a single data point requires 0.025 ms. The grid refinement minimization of the optimization function consists of 3 iterations with a net of 40 temperature times 200 magnetic field points within the measurement range. The calculation for a single data point requires 70 ms. Calculation times are not optimized.
